# Mesenchymal Stem Cells Induce T-Cell Tolerance and Protect the Preterm Brain after Global Hypoxia-Ischemia

**DOI:** 10.1371/journal.pone.0073031

**Published:** 2013-08-26

**Authors:** Reint K. Jellema, Tim G. A. M. Wolfs, Valéria Lima Passos, Alex Zwanenburg, Daan R. M. G. Ophelders, Elke Kuypers, Anton H. N. Hopman, Jeroen Dudink, Harry W. Steinbusch, Peter Andriessen, Wilfred T. V. Germeraad, Joris Vanderlocht, Boris W. Kramer

**Affiliations:** 1 School for Mental Health and Neuroscience, Maastricht University, Maastricht, The Netherlands; 2 Department of Pediatrics, Maastricht University Medical Center, Maastricht, The Netherlands; 3 School of Oncology and Developmental Biology, Maastricht University, Maastricht, The Netherlands; 4 Department of Methodology & Statistics, Maastricht University, Maastricht, The Netherlands; 5 Department of Biomedical Engineering, Maastricht University, Maastricht, The Netherlands; 6 Department of Molecular Cell Biology, Maastricht University, Maastricht, The Netherlands; 7 Department of Internal Medicine, Division of Haematology, Maastricht University Medical Center, Maastricht, The Netherlands; 8 Department of Transplantation Immunology, Tissue Typing Laboratory, Maastricht University Medical Center, Maastricht, The Netherlands; 9 Department of Neonatology and Neuroscience, Sophia Children’s Hospital, Rotterdam, The Netherlands; 10 Department of Pediatrics, Máxima Medical Centre, Veldhoven, The Netherlands; University of South Florida, United States of America

## Abstract

Hypoxic-ischemic encephalopathy (HIE) in preterm infants is a severe disease for which no curative treatment is available. Cerebral inflammation and invasion of activated peripheral immune cells have been shown to play a pivotal role in the etiology of white matter injury, which is the clinical hallmark of HIE in preterm infants. The objective of this study was to assess the neuroprotective and anti-inflammatory effects of intravenously delivered mesenchymal stem cells (MSC) in an ovine model of HIE. In this translational animal model, global hypoxia-ischemia (HI) was induced in instrumented preterm sheep by transient umbilical cord occlusion, which closely mimics the clinical insult. Intravenous administration of 2 x 10^6^ MSC/kg reduced microglial proliferation, diminished loss of oligodendrocytes and reduced demyelination, as determined by histology and Diffusion Tensor Imaging (DTI), in the preterm brain after global HI. These anti-inflammatory and neuroprotective effects of MSC were paralleled by reduced electrographic seizure activity in the ischemic preterm brain. Furthermore, we showed that MSC induced persistent peripheral T-cell tolerance *in vivo* and reduced invasion of T-cells into the preterm brain following global HI. These findings show in a preclinical animal model that intravenously administered MSC reduced cerebral inflammation, protected against white matter injury and established functional improvement in the preterm brain following global HI. Moreover, we provide evidence that induction of T-cell tolerance by MSC might play an important role in the neuroprotective effects of MSC in HIE. This is the first study to describe a marked neuroprotective effect of MSC in a translational animal model of HIE.

## Introduction

Preterm infants are prone to brain injury after a perinatal hypoxic-ischemic insult [1–3]. Hypoxic-ischemic encephalopathy (HIE) in preterm infants is predominantly characterized by white matter injury (i.e. periventricular leukomalacia) which is caused by damage to highly vulnerable immature oligodendrocytes [1,2,4]. HIE in preterm infants is associated with cognitive disorders in 25-50% of all cases and 5-10% suffer from severe motor deficits (i.e. cerebral palsy) [5]. However, therapeutic options to improve the neurodevelopmental outcome in preterm infants after HIE are unavailable.

There is mounting evidence that the inflammatory response following brain ischemia plays a crucial role in the pathophysiology of ischemic brain injury [6,7]. This concept is predominantly based on literature showing activation of the cerebral and peripheral immune system after focal ischemia (i.e. stroke; transient or permanent occlusion of cerebral perfusion) in adult [8,9] and term neonatal [10] rodent models. Recently, we have demonstrated in a translational ovine model, that global hypoxia-ischemia (HI), which was induced by transient umbilical cord occlusion, caused cerebral inflammation and activation of the peripheral immune system in a similar way as observed after focal ischemia [11]. More precisely, we showed in this model, which is representative for brain development of preterm infants, that global HI induced a profound microglial response followed by a second peripheral inflammatory response characterized by invasion of mobilized peripheral immune cells into the ischemic preterm ovine brain [11]. These inflammatory changes were associated with marked injury to pre-oligodendrocytes and hypomyelination of the preterm brain [11], which are well known indicators of white matter injury in the ischemic preterm brain [1,2,12]. Our findings indicated that the immature immune system is readily mobilized after global HI and is involved in the etiology of white matter injury, the clinical hallmark of hypoxic-ischemic preterm brain injury [11].

Since inflammation plays an important role in the etiology of neonatal brain injury, neuroprotective therapies should have strong anti-inflammatory and regenerative capacities if aimed at the repair of the hypoxic-ischemic neonatal brain. Mesenchymal stem cells (MSC) meet these criteria [13–16], and therefore several studies have been conducted to assess whether MSC therapy can protect the neonatal term brain after focal ischemia [17–22]. The objective of our study was to assess the neuroprotective and anti-inflammatory potential of MSC therapy in the preterm brain exposed to global hypoxic-ischemia. We hypothesized that intravenously administered human bone-marrow derived MSC would be neuroprotective in a translational animal model of preterm HIE. To test this hypothesis, preterm instrumented sheep were exposed to 25 minutes of umbilical cord occlusion at 0.7 gestation. At this time of gestation neurodevelopment of fetal sheep is equivalent to that of a preterm infant of 30-32 weeks [23,24]. The neuroprotective effect of MSC treatment was studied by assessment of white matter injury and electrographic seizure activity. The anti-inflammatory effect of MSC treatment was studied by evaluation of the peripheral T-cell response.

## Materials and Methods

### Ethics Statement

The experimental protocol and design of the study were in line with the institutional guidelines for animal experiments and were approved by the institutional Animal Ethics Research committee of Maastricht University, The Netherlands.

### Animals and surgery

Fetuses of time-mated Texel ewes were instrumented as previously described [11]. In short, all fetuses were instrumented with an arterial catheter for blood pressure measurements and blood sampling, a venous catheter for administration of MSC and ECG electrodes for heart rate monitoring. EEG electrodes were placed as previously described [11]. The anterior and posterior placed electrodes were considered C_3_–C_4_ channel and P_3_–P_4_ channel, respectively. An inflatable vascular occluder (OC16HD, 16mm, In Vivo Metric, Healdsburg, CA, USA) was placed around the umbilical cord to induce transient global HI. All fetal catheters and leads were exteriorized through a trocar hole in the flank of the ewe. The welfare of the animals was monitored daily by certified personnel.

### Randomization and blinding

**Figure 1:**
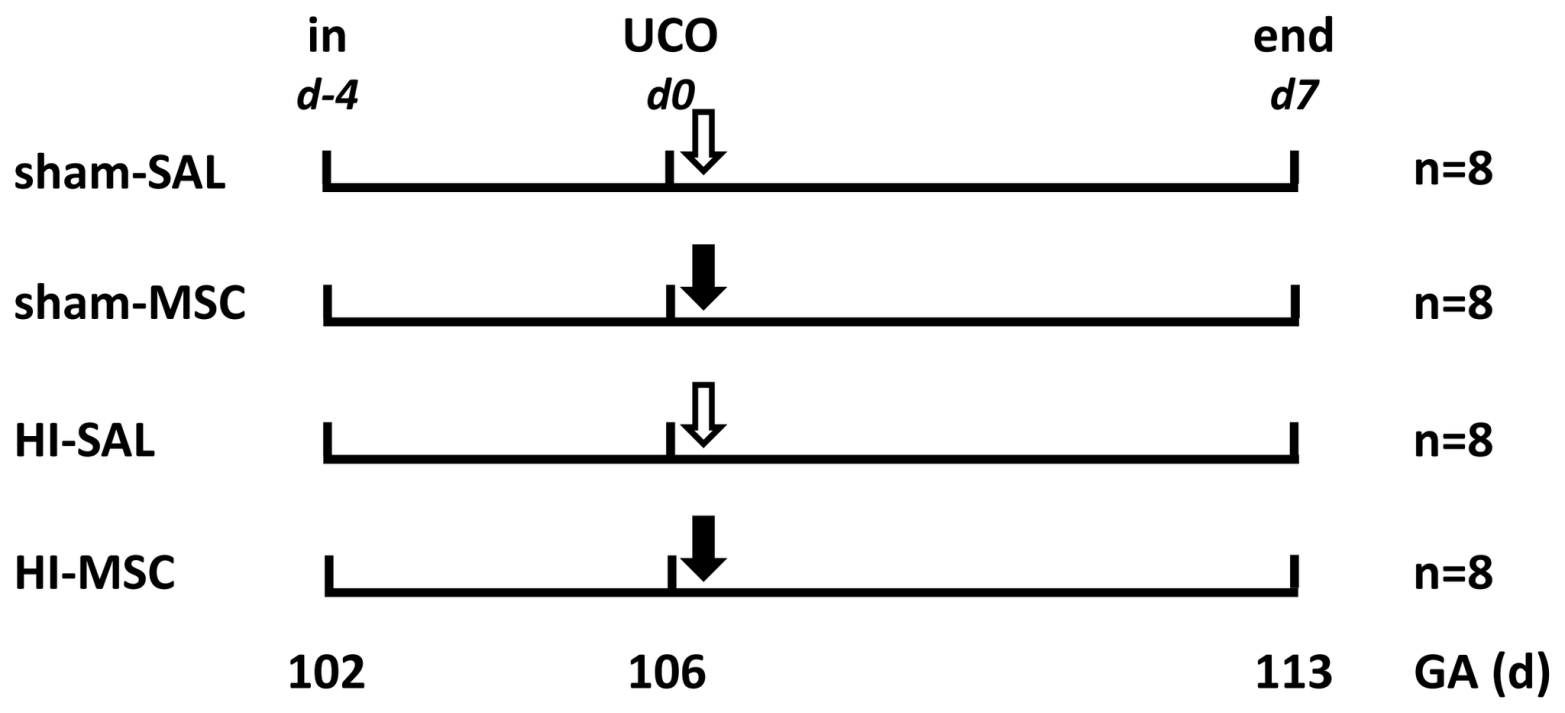
Study design. Fetuses were instrumented at a gestational age (GA) of 102 d. After a recovery period of 4 d fetuses were subjected to 25 min of umbilical cord occlusion (UCO) or sham. One hour after UCO or sham, fetuses received either intravenous mesenchymal stem cells (2.0 million/kg BW) (closed arrow) or saline 0,9% (open arrow). After a 7 d reperfusion period brain tissue was collected. Abbreviations: in = instrumentation, HI = hypoxia-ischemia, SAL = saline, MSC = mesenchymal stem cells.

Prior to the entire series of experiments, animals were randomized by an independent researcher who was not involved in the animal experiments. The randomization resulted in four experimental groups (Figure 1). The investigator performing the (sham) umbilical cord occlusions was blinded for treatment allocation. Tissue sampling and the analyses of brain tissue and electrophysiological data were conducted in a blinded fashion.

### Experimental design

**Figure 2:**
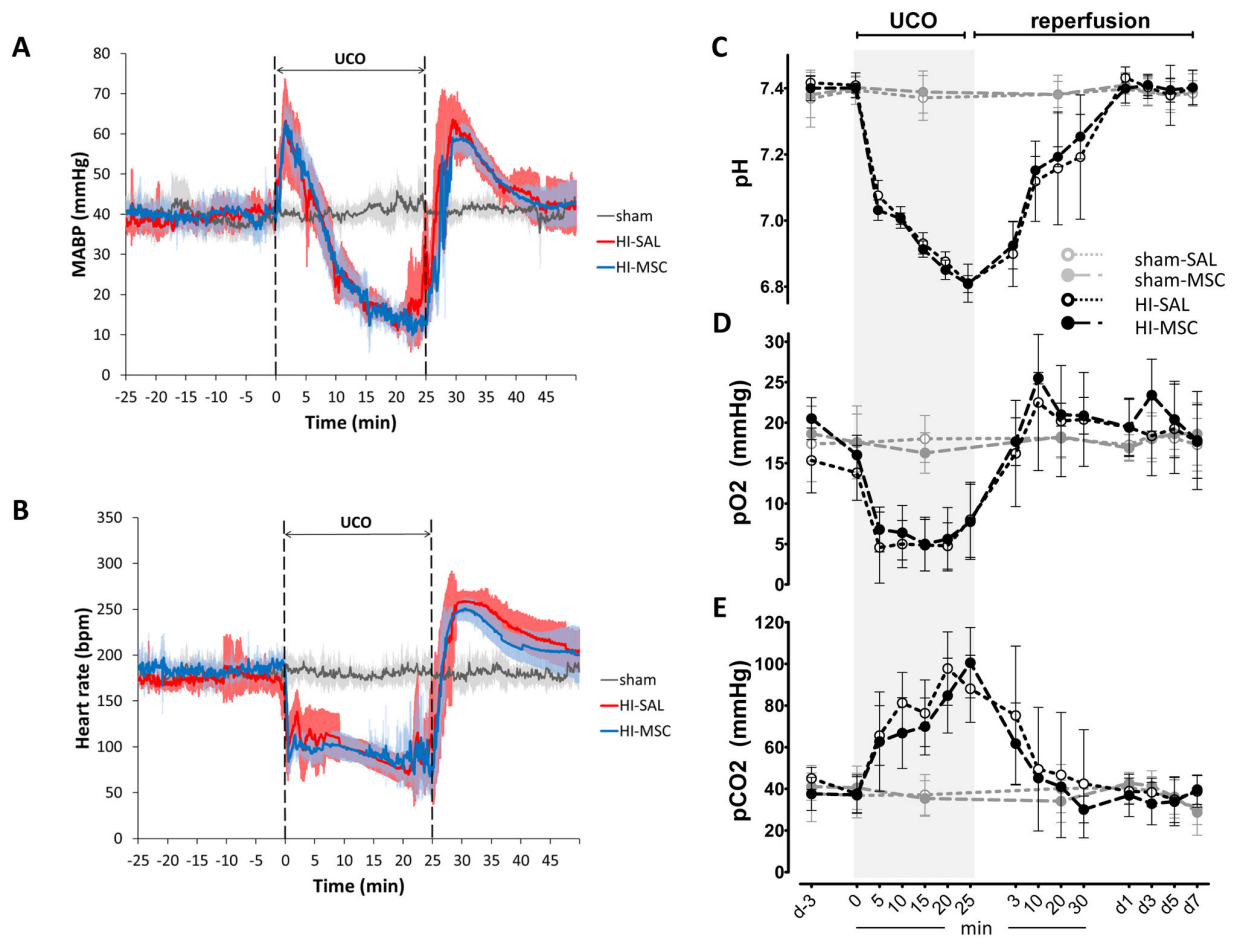
Reproducibility of 25 min umbilical cord occlusion (UCO). (**A**) Fetal mean arterial blood pressure (MABP) and (**B**) fetal heart rate (FHR) measurements indicated that all animals exposed to global HI experienced the same degree of hypotension and bradycardia, respectively, at the end of UCO; means (thick line) ± SD (shaded areas) of n=8 animals per experimental group are shown. MABP and FHR normalized within one hour after the end of UCO and were stable throughout the rest of the study period. All sham operated animals had similar MABP and FHR parameters during the entire duration of the study. For clarity reasons the sham-SAL and sham-MSC groups are depicted as one sham group. (**C**–**E**) Blood gas analysis indicated that twenty five minutes of umbilical cord occlusion induced comparable acidosis, hypoxemia and hypercarboxemia in all animals exposed to global HI, as demonstrated by (**C**) arterial pH, (**D**) arterial partial oxygen pressure (pO_2_) and (**E**) arterial partial carbon dioxide pressure (pCO_2_), respectively; means (lines) ± SD (error bars) of n=8 animals are depicted. Blood gas indices in animals exposed to global HI normalized within one hour after the end of UCO and stayed within the normal range throughout the rest of the study period.

Fetuses were instrumented at 101.5 ± 1.0 (mean ± SD) days of gestation (experimental day -4). After surgery ewe and fetus were allowed to recover for four days. At gestational age 105.5 ± 1.1 (mean ± SD) (experimental day 0) fetuses were subjected to 25 minutes of umbilical cord occlusion (Figure 2) by rapidly inflating the occluder. After umbilical cord occlusion a reperfusion period of 7 d followed.

### Mesenchymal stem cells

Human bone marrow-derived mesenchymal stem cells (Merck Millipore, Billerica MA, USA) isolated from a male healthy donor were expanded following the manufacturer’s protocol. After four passages the cells were frozen in freezing medium containing 10% FCS and 10% DMSO and stored in liquid nitrogen. The expanded MSC were validated for their differentiation potential and cell surface molecules. MSC effectively differentiated into osteoblasts and adipocytes (data not shown). Expression of cell surface molecules on infused MSC was confirmed by flow cytometry analysis. MSC expressed CD44, HLA-I, CD49c, CD13, CD54, CD58, CD140b, CD105, CD90, CD73 dimly expressed CD117 and CD49d and did not express HLA-II, CD45, CD34, CD40, CD80, CD146, CD3, CD86 and CD19.

MSC were thawed and washed twice with PBS one hour before administration. MSC were infused intravenously 1 h after (sham) umbilical cord occlusion. Fetuses received 3.5 x 10^6^ MSC (approximately 2.0 x 10^6^/kg BW) resuspended in 1 mL PBS via the venous line.

### Data acquisition

Physiological data was acquired as described previously [11]. After filtering, the raw EEG signals of the central and posterior channels were converted into amplitude-integrated EEG (aEEG) traces, using a Matlab^®^ (R2011b; The Mathworks Inc., Natick, MA, USA) algorithm similar to the clinical EEG NicoletOne^TM^ device (Viasys Healthcare, Conshohocken, PA, USA) [25]. The EEG processing included an asymmetric band-pass filter that strongly attenuates activity below 2 Hz and above 15 Hz, semi-logarithmic amplitude compression and time compression [26]. The (a) EEG traces were used to detect electrographic seizure activity, which in the aEEG usually is seen as an abrupt rise in the lower margin amplitude (LMA) and a simultaneous rise in the upper margin amplitude (UMA), often followed by a short period of decreased amplitude [27]. The raw EEG simultaneously shows gradual build-up and decline in amplitude of repetitive sharp-waves [28]. From 24 h before UCO to the end of the experiment, aEEG and EEG were displayed simultaneously. Electrographic seizure activity with a duration ≥ 10 s was annotated using both aEEG/EEG traces in n=6 animals per experimental group. Annotation was performed by a neonatologist, experienced in neonatal aEEG interpretation, who was blinded for treatment allocation.

### MRI

At the end of the experiment (day 7), the fetal brain was removed from the skull and weighed. The right hemisphere was submersion fixated in 4% paraformaldehyde (PFA) for 3 months and stored at 4 degrees Celsius. Forty eight hours prior to MRI imaging, the right hemisphere was washed with PBS and stored in PBS containing 1% sodiumazide at 4 degrees Celsius to wash out all PFA. After optimization, all DTI images were acquired using an echo planar imaging (EPI) sequence with diffusion gradients (b=4000 s/mm^2^) applied in 66 non-collinear directions and 6 B0 measurements. An average of 60 slices was recorded within 36 minutes using a repetition time (TR) = 500 ms and echo time (TE) = 75 ms. Isovolumetric voxel size was 0.5 mm^3^. The FOV was 30x60x60 mm and scan matrix size 60x120x60 mm.

DTI data was post processed using ExploreDTI software and fractional anisotropy (FA) maps maps were displayed [29]. White matter injury was assessed by measuring fractional anisotropy (FA), which has been previously shown to be unaffected by fixation [30]. Delineation of ROIs was performed by a neonatologist, experienced in DTI analysis, who was blinded for the treatment allocation. FA values within the delineated ROI in the SCWM and hippocampus were calculated with ExploreDTI [29].

### Immunohistochemistry brain

Following MRI imaging the right hemisphere was embedded in gelatin and serial coronal sections (50 µm) were cut on a Leica VT 1200S vibrating microtome (Leica Biosystems, Nussloch, Germany). Free floating sections at the level of mid-thalamus and posterior hippocampus were stained with a rabbit anti-ionized calcium binding adaptor molecule 1 (IBA-1) antibody (Wako Pure Chemical Industries, Osaka, Japan), which is a highly specific marker for microglia, localizing resting and activated microglia [11]. A mouse anti-O4 antibody (Merck Millipore, Billerica, MA, USA) was used to detect pre-oligodendrocytes (preOLs) (oligodendrocyte progenitors and immature oligodendrocytes) and a rat anti-myelin basic protein (MBP) antibody (Merck Millipore, Billerica, MA, USA) was used to detect myelin sheaths and myelin producing (mature) oligodendrocytes. Endogenous peroxidase-activity was blocked by incubation with 0,3% H_2_O_2_ in Tris buffered saline (TBS, pH 7.6). Free floating sections were incubated overnight (anti-IBA-1 and MBP) or during three days (anti-O4) at 4°C with the diluted primary antibody (1:1000 anti-IBA-1, 1:400 anti-O4 and 1: 2000 MBP) followed by incubation with a secondary donkey-anti-rabbit (anti-IBA-1), donkey-anti-rat (MBP) or donkey-anti-mouse (anti-O4) biotin labeled antibody. The immunostaining was enhanced with Vectastain ABC peroxidase Elite kit (PK-6200, Vector Laboratories, Burlingame, CA, USA) followed by a nickel sulfate-diaminobenzidine (NiDAB) staining. Sections were mounted on gelatin-coated glass slides, air-dried, dehydrated in ascending ethanol concentrations and coverslipped with PerTex.

After cutting of free floating sections, the remainder of the tissue was embedded in paraffin and serial coronal sections (4 µm) were cut. Sections at the level of mid-thalamus and posterior hippocampus were stained with CD3 (DAKO A0452, DAKO, Denmark) for detection of T-cells. Endogenous peroxidase was inactivated by incubation with 0,3% H_2_O_2_ that was dissolved in PBS. Antigen retrieval was performed by boiling in citrate buffer pH 6.0. Antigen aspecific binding was prevented by incubating the slides for 30 minutes with 5% bovine serum albumin (BSA). Slides were incubated overnight at 4°C with the diluted primary antibody (CD3 1:200) followed by incubation with the appropriate secondary biotin labeled antibody. Immunostaining was enhanced with Vectastain ABC peroxidase Elite kit (PK-6200, Vector Laboratories) followed by a NiDAB staining. Sections were counterstained with 0.1% Nuclear Fast Red washed, dehydrated and coverslipped.

### Analysis of immunohistochemistry

**Figure 3:**
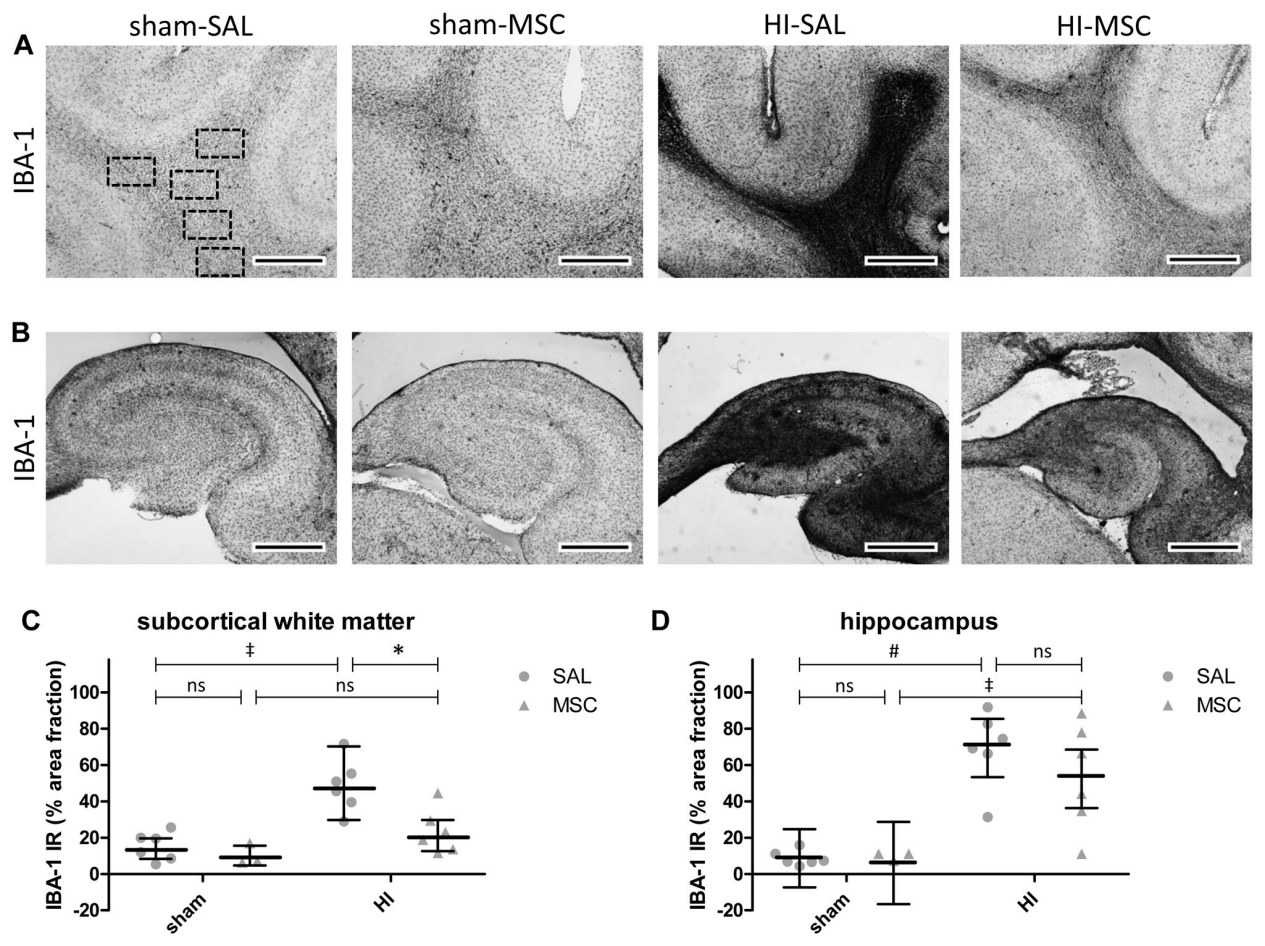
Intravenous MSC reduced proliferation of microglia after global HI. (**A**) Immunohistochemical IBA-1 staining in the SCWM of the four experimental groups with squares in the first panel indicating the regions where immunoreactivity was assessed. Global HI induced a profound increase of IBA-1 immunoreactivity, which was significantly reduced by intravenous MSC treatment. (**B**) Immunohistochemical IBA-1 staining in the hippocampus of the four experimental groups. Profound proliferation of microglia was observed in the hippocampus following global HI. MSC partially reduced the inflammatory response of microglia in the hippocampus after global HI. (**C**–**D**) Graphical presentation of area fraction of IBA-1 immunoreactivity in SCWM and hippocampus; (**C**) geometric means ± 95% CI and (**D**) means ± 95% CI and levels of significance are depicted, which were calculated by the random intercept model with all repeated measures (i.e. brain sections) per animal (sham-SAL n=6, sham-MSC n=3, HI-SAL n=6, HI-MSC n=6). Dots show the averaged results of the repeated measures (i.e. brain sections) per animal. * *P*≤0.05, ‡ *P*≤0.01, # *P*≤0.001. IBA-1 = ionized calcium binding adaptor molecule 1, HI = hypoxia-ischemia, SAL = saline, MSC = mesenchymal stem cells, IR = immunoreactivity. (**A**–**B**) Scale bars represent 1 mm.

For the analysis of IBA-1 immunoreactivity, digital images of the subcortical white matter (SCWM) (100x magnification) and hippocampus (20x magnification) were acquired using an Olympus AX-70 microscope (Olympus, Tokyo, Japan) equipped with a black and white digital camera. For the analysis of IBA-1 immunoreactivity, n=6 for sham-SAL, n=3 for sham-MSC, n=6 for HI-SAL and n=6 for HI-MSC animals per group were analyzed. In each individual animal, IBA-1 immunoreactivity was assessed in six consecutive coronal sections (posterior hippocampus/ mid-thalamus level). In the hippocampus, area fraction of IBA-1 immunoreactivity was assessed in one 20x digital image per section by delineating the hippocampus and determining the areal fraction of IBA-1 immunoreactivity expressed as a percentage of total hippocampal area with a standard threshold using Leica Qwin Pro V 3.5.1 software (Leica, Rijswijk, the Netherlands). In the SCWM, the area fraction of IBA-1 immunoreactivity was determined in five adjacent 100x digital images obtained in standardized locations within the SCWM of each section (Figure 3A). The results of five images per section were averaged to obtain the areal fraction of IBA-1 immunoreactivity within the SCWM for each section. The digital images were obtained and analyzed by an independent observer who was blinded to the experimental conditions.

**Figure 4:**
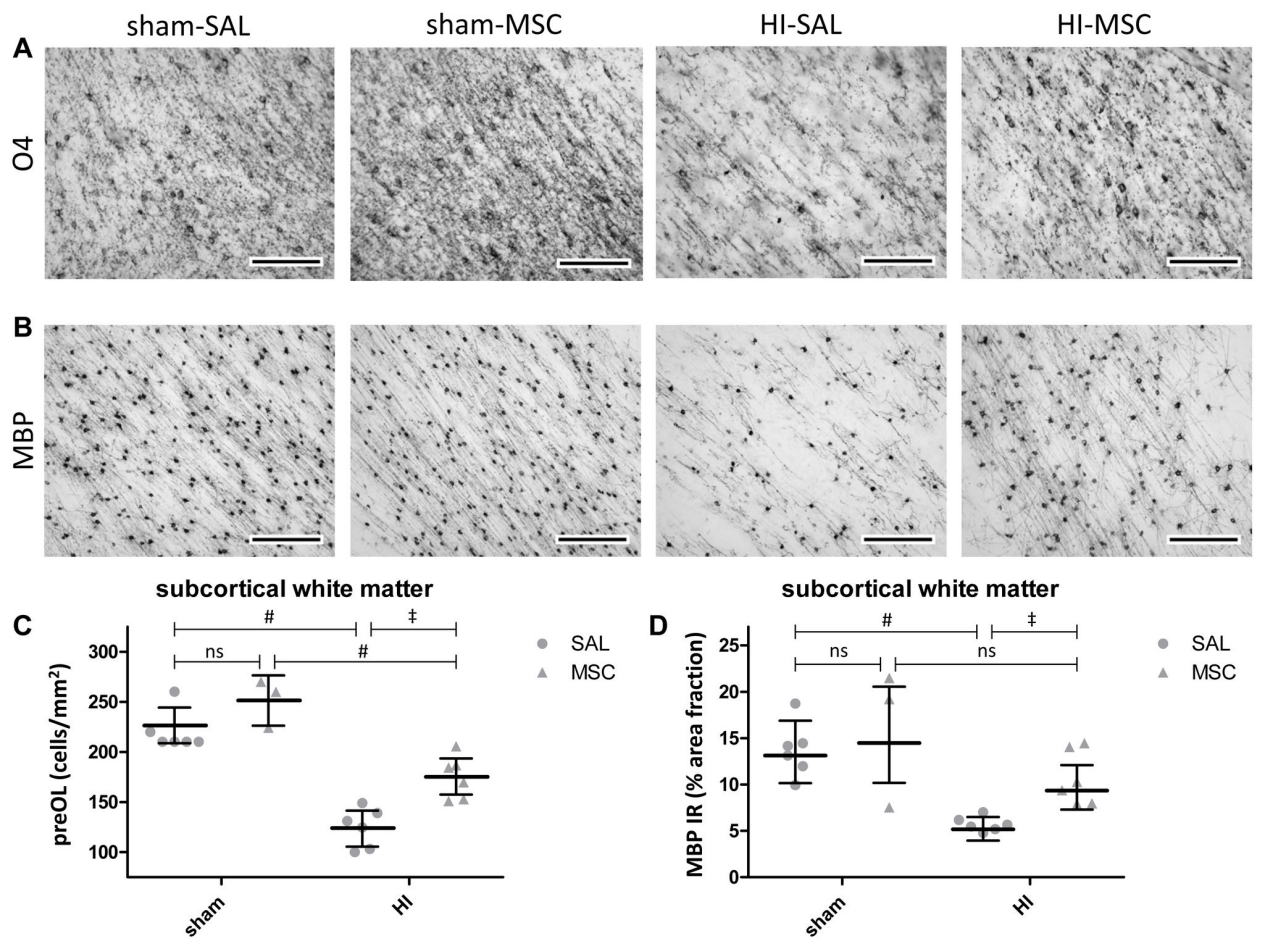
Intravenous MSC treatment reduced loss of pre-oligodendrocytes and demyelination after global HI. (**A**) Immunohistochemical O4 staining in the subcortical white matter (SCWM) of all four experimental groups. The number of O4-positive cells was similar between sham operated animals treated with saline or MSC. Global HI induced marked loss of O4-positive cells. Intravenous MSC significantly increased the number of O4-positive cells after global HI. (**B**) Immunohistochemical MBP staining in the SCWM of all four experimental groups. The area fraction of MBP was similar between sham operated animals treated with saline or MSC. Marked hypomyelination was observed in the SCWM following global HI. Intravenous MSC significantly increased myelination of the preterm brain after global HI. (**C**–**D**) Graphical presentation of number of O4-positive cells and MBP immunoreactivity in SCWM; (**C**) means ± 95% CI (**D**) geometric means ± 95% CI and levels of significance are depicted, which were calculated by the random intercept model with all repeated measures (i.e. brain sections) per animal (sham-SAL n=6, sham-MSC n=3, HI-SAL n=6, HI-MSC n=6). Dots show the averaged results of the repeated measures (i.e. brain sections) per animal. * *P*≤0.05, ‡ *P*≤0.01, # *P*≤0.001. PreOL = pre-oligodendrocytes, HI = hypoxia-ischemia, SAL = saline, MSC = mesenchymal stem cells, IR = immunoreactivity, log = natural logarithm. Scale bars: (**A**) 100 µm, (**B**) 200 µm.

For the analysis of MBP immunoreactivity, digital images of the subcortical white matter (SCWM) (100x magnification) were acquired using the Olympus AX-70 microscope. For the analysis of MBP immunoreactivity, n=6 for sham-SAL, n=3 for sham-MSC, n=6 for HI-SAL and n=6 for HI-MSC animals per group were analyzed. In each individual animal, MBP immunoreactivity was assessed in six consecutive coronal sections (posterior hippocampus/ mid-thalamus level). In the SCWM, the MBP immunoreactivity was determined in five adjacent 100x digital images obtained in standardized locations within the SCWM of each section (Figure 3A). Areal fraction of MBP-positive myelin sheaths was determined in each digital image with a standard low-pass intensity threshold set to detect all MBP immunoreactivity and a standard high-pass intensity threshold set to exclude the intense staining of the MBP-positive mature oligodendrocytes (Figure 4B) using Leica Qwin Pro V 3.5.1 software. By excluding the immunoreactivity of MBP-positive mature oligodendrocytes the measurements accurately represented white matter only. The results of five images per section were averaged to obtain the areal fraction of MBP immunoreactivity within the SCWM for each section. The digital images were obtained and analyzed by an independent observer who was blinded to the experimental conditions.

O4 immunoreactivity in the SCWM was assessed as previously described [11]. In short, a differential count was performed, discriminating between immature (ring-shaped membrane staining, no processes), mature (ring-shaped membrane staining, extensively branched processes) and degenerative (fragmented membrane staining, fragmentation of processes, signs of cell death; nuclear condensation and apoptotic bodies) phenotype of the O4 positive cells. The sum of the differential count resulted in the total number of O4 positive cells. For the analysis of O4 positive cells in the SCWM, n=6 for sham-SAL, n=3 for sham-MSC, n=6 for HI-SAL and n=6 for HI-MSC animals per group were analyzed. In each individual animal, the number of O4 positive cells was assessed in six consecutive coronal sections (posterior hippocampus/ mid-thalamus level). In each section, O4 positive cells were counted in eight randomly chosen fields of view in the SCWM (within the regions as indicated in Figure 3A) with a 40x objective equipped with a counting grid (0.0625 mm^2^) using a Nikon Eclipse E400 microscope (Nikon, Amsterdam, the Netherlands). The results of the eight differential counts were averaged to obtain the differential count of O4 positive cells per section. The investigator who performed the differential count was blinded to the experimental conditions.

For the analysis of CD3 staining in the SCWM, three coronal sections per animal (n=4 for sham-SAL, n=4 for sham-MSC, n=4 for HI-SAL and n=4 for HI-MSC) were studied at the posterior hippocampus/ mid-thalamus level. In each section, cells were counted in ten fields of view (focused on the cerebral vasculature) in the subcortical white matter with a 20x objective equipped with a counting grid (0.25 mm^2^) using a Nikon Eclipse E400 microscope (Nikon, Amsterdam, the Netherlands). The numbers of CD3-positive cells were expressed as cells per field of view (FOV).

### Proliferation assay

At the end of the experiment (day 7), the spleen was immediately harvested after sacrifice. Single-cell splenocyte suspensions were obtained by dissociating freshly sampled spleen tissues in gentleMACS™ C-tubes (MiltEnyi, Leiden, the Netherlands) filled with Gibco® Iscove’s Modified Dulbecco’s Medium (IMDM) (Life Technologies, Bleiswijk, the Netherlands) using the gentleMACS™ Dissociator (MiltEnyi, Leiden, the Netherlands). Subsequently, the cell suspensions were passed through a 70 µm cell strainer (BD Biosciences, Erembodegem-Aalst, Belgium). Splenocytes were stored in nitrogen in freezing medium containing IMDM medium with 10% heat-inactivated FCS and 10% DMSO.

For the proliferation assay, splenocytes of all experimental groups (sham-SAL n=4, sham-MSC n=4, HI-SAL n=4, HI-MSC n=4) were thawed. Splenocytes were labeled with CFSE (Sigma-Aldrich, Zwijndrecht, The Netherlands) in a final concentration of 1 µM. CFSE-labeled splenocytes were plated at a concentration of 1.0 million cells/mL in a round bottom 96-wells plate in DMEM (Gibco®, Life Technologies, Bleiswijk, The Netherlands) culture medium containing 10% FCS and 1% Penicillin Streptomycin solution. CFSE-labeled splenocytes were stimulated by adding Phytohemagglutinin (PHA) (Lectin; Sigma-Aldrich, Zwijndrecht, The Netherlands) at a final concentration of 1 µg/mL and human recombinant IL-2 (Proleukin®; Aldesleukin (Chiron), Novartis, Basel, Switzerland) at a final concentration of 500 IU/mL to the culture medium. These final concentrations yielded optimal proliferation of fetal sheep splenocytes in pilot experiments (data not shown). Stimulated splenocytes were cultured during five days in an incubator set at 37°C and 5% CO_2_. On day 5 the proliferated splenocytes were stained for detection of T-helper cells (mouse anti sheep CD4-AlexaFluor® 647 (CD4-A647); AbDSerotec, Düsseldorf, Germany), cytotoxic T-cells (mouse anti sheep CD8-R-PE (CD8-PE); AbDSerotec, Düsseldorf, Germany) and viability (7-Aminoactinomycin D (7-AAD); BD Biosciences, Bleiswijk, the Netherlands) according to the manufacturer’s protocol. Stained cells were acquired on a FACS Canto II flow cytometer (BD Biosciences, Bleiswijk, the Netherlands) equipped with FACS Diva software (BD Biosciencs, Bleiswijk, the Netherlands). Gating strategy is depicted in Figure S1A–C. The number of non-proliferating (high CFSE fluorescent intensity) CD4- and CD8-positive cells was expressed as a percentage of total viable splenocytes in the well.

Because of the lack of good flow cytometry antibodies cross-reacting with ovine lineage markers it was not possible to gate on CD3. However, it was possible to discriminate between ovine CD4-positive T-cells and CD8-positive T-cells (gating strategy; Figure S1). Cells being positive for both CD4 and CD8 are presumably monocytes or myeloid cells (with Fc receptor) and were not taken along in our analysis. The gate for the non-proliferating fraction was set based on the condition without mitogen (data not shown).

### Fluorescent In Situ Hybridization (FISH)

The FISH probe for chromosomes Y (DYZ3, Sat. III) [31] was selected to identify intravenously administered human male MSC in the preterm ovine brain. The probe was labeled by standard nick translation with biotin (Bio). The labeled probe was applied at a concentration of 1 ng/µl in 60% formamide, 2x SSC, 10% dextran sulphate, and a 50x excess of carrier DNA (salmon sperm DNA). FISH was performed on 4 µm thick formalin fixed and paraffin embedded tissue sections (at the posterior hippocampus/ midthalamus level) put on amino coated slides (superfrost plus+). In brief, sections were heated at 80 °C for 15 minutes to improve cellular adhesion to the slides during the entire FISH procedure, dewaxed and hydrated followed by microwaving in 10 mM Na-Citrate pH buffer 1 x 10 min at 100 °C and 20 min at RT to cool down. Washed in demineralized water and rinsed in 0.01 M HCl and subsequently digested in 2.5 mg pepsin in 0.01 N HCl. Afterwards the slides were washed in 1 x 0.01 N HCl, 1 x PBS and post fixed in 1% formaldehyde in PBS for 5 min at RT. Slides were washed with PBS, demineralized water and dehydrated in an alcohol series. The probe was applied under a coverslip, simultaneously denatured at 80 °C for 10 min and hybridized overnight at 37 °C. After hybridization, the preparations were washed for 5 min in 2 x SSC, 0.05% tween-20 (Janssen Chimica, Beerse, Belgium) and 0.1 X SSC 61 °C (2 × 5 min). The hybridized probe was detected in a triple layer detection method with FITC-conjugated avidin (Av-FITC, 1: 100 dilution; Vector Laboratories, CA, USA), biotinylated goat anti-avidin (Bio-GaA, 1:100 dilution: Vector Laboratories) and Av-FITC. Finally, the slides were washed in PBS containing 0.05% Tween-20, dehydrated in an ascending ethanol series, and mounted in Vectashield (Vector Laboratories) containing DAPI (Sigma: 0.5 µg/µl). Images were recorded with the Metasystems Image Pro System (black and white CCD camera; Sandhausen, Germany) mounted on top of a Leica DM-RE fluorescence microscope equipped with FITC and DAPI single band pass filters for single color analysis. Images were recorded using an automatic integration time allowing semi quantitative evaluation (using the full dynamic range of the camera without signal intensity saturation; TIF 8 bits image).

### PCR

The distribution of MSC throughout the tissues was confirmed by PCR for human-specific β-2 microglobulin as previously reported [32]. Genomic DNA that was used as template was extracted from snap frozen tissues including subcortical white matter and hippocampus of the left hemisphere, fetal lung (right middle lobe) and spleen using the Wizard Genomic Purification kit (Promega, Leiden, The Netherlands) according to the supplier’s recommendations. The presence of amplifiable DNA was evaluated by PCR for β-actin. PCR reactions were performed in a total volume of 25 µl with specific primers for β-actin or β-2 microglobulin using 1 µl of genomic DNA. Next, a nested PCR was conducted for β-2 microglobulin using 2.5 µl of the amplified product of the first reaction as a template. The amplified products were separated on ethidium bromide-stained 2% agarose gels and captured using the Imagemaster® VDS equipped with a CCD camera (GE Healthcare Life Sciences (Pharmacia Biotech), Uppsala, Sweden). The amplified products were evaluated using the Big Dye termination sequencing kit (PerkinElmer/Cetus, Emeryville, CA).

Primers for β-actin were 5’-CGGGACCTGACTGACTAC-3’ (sense) and 5’-GAAGGAAGGCTGGAAGAG-3’ (antisense); primers used for the amplification of β-2 microglobulin were 5’- GTGTCTGGGTTTCATCAATC-3’ (sense), 5’- GGCAGGCATACTCATCTTTT-3’ (antisense), 5’- TGGGTTTCATCAATCCGACAT-3’ (nested sense) and 5’- CTCATCTTTTTCAGTGGGGGT-3’ (nested antisense).

PCR were performed in a total volume of 25 µl in PCR buffer (Promega, Leiden, The Netherlands) in the presence of 0.2 mM dNTP (Promega, Leiden, The Netherlands), 1 M of each primer, 0.3 mM MgCl2, and 0.5 U of Taq polymerase (Promega, Leiden, The Netherlands). PCR conditions for each primer couple were as follows: β-actin; 95°C for 30 s, 53°C for 45 s and 72°C for 30 s during 40 cycles: human-specific β-2 microglobulin; 95°C for 30 s, 53°C for 30 s and 72°C for 20 s during 45 cycles: human-specific β-2 microglobulin nested 95°C for 30 s, 50°C for 45 s and 72°C for 20 s during 45 cycles.

### Statistics

Summary statistics of animal characteristics and all outcome parameters are shown as means with 95% CI. Groups’ comparisons with respect to all outcome parameters (except seizure data) were drawn with analysis of variance (ANOVA) or with random intercept models in case of repeated measurements per animal (e.g. different sections per brain). Since these tests assume normal distribution of the data, variables, whose distributions were positively skewed, were log-transformed previous to statistical testing. To facilitate interpretation, averages on the log scale were back transformed to the original scale (antilog) and are presented as geometric means and corresponding 95% CIs, as previously reported [11]. Seizure data was zero-inflated (i.e. no seizures in sham animals), showing pronounced skewness that could not be remedied by log transformation. Hence, pair-wise groups’ comparisons of seizure data were performed with nonparametric Mann-Whitney tests. Seizure data are presented as medians and corresponding interquartile range (IQR). For assessment of correlations, the Pearson or Spearman correlation coefficient was calculated, as appropriate. To avoid overestimation of treatment effects, a False Discovery Rate (FDR) of 5% was used for multiple testing correction. Groups’ differences with FDR corrected P<0.05 were considered statistically significant. Statistical analysis was performed with IBM SPSS Statistics Version 20.0 (IBM Corp., Armonk, NY, USA).

## Results

### Animal characteristics and reproducibility of umbilical cord occlusion

Fetal body weight and gestational age at the time of UCO did not differ significantly between the four experimental groups (Table 1). No differences were found in MABP, FHR, pH, arterial partial oxygen pressure or arterial partial carbon dioxide pressure between the HI-SAL and HI-MSC group, indicating that the physiological response to UCO and the degree of acidosis, hypoxemia and hypercarboxemia was similar in all animals exposed to global HI (Figure 2).

**Table 1:** Animal characteristics.

	**sham-SAL (n=8)**	**sham-MSC (n=8)**	**HI-SAL (n=8)**	**HI-MSC (n=8)**
	mean (95% CI)	mean (95% CI)	mean (95% CI)	mean (95% CI)
GA at UCO (d)	105.5 (104.9;106.1)	105.3 (104.6;106.1)	105.3 (104.7;105.9)	105.8 (105.0;106.7)
BW (g)	1791.9 (1602.2;1963.7)	1783.0 (1584.7;1981.3)	1742.0 (1553.8;1930.2)	1829.7 (1586.8;2072.6)
brain (g)	29.9 (28.2;31.5)	29.0 (27.3;30.8)	24.7 (23.3;26.2)	27.5 (25.5;29.5)

Fetuses were subjected to umbilical cord occlusion at a comparable age. Fetal body weight did not differ between experimental groups. Global HI caused significant brain atrophy, which was prevented by intravenous MSC administration.

### MSC reduced microglial proliferation after global HI

Global HI resulted in a significant (sham-SAL vs. HI-SAL; P=0.002) increase of IBA-1 immunoreactivity in the SCWM, indicating profound microglial proliferation in this region (Figure 3 A and C). In line, significantly (sham-SAL vs. HI-SAL; P<0.001) increased IBA-1 immunoreactivity was found in the hippocampus following global HI (Figure 3 B and D). Intravenous MSC significantly (HI-SAL vs. HI-MSC; P=0.020) reduced IBA-1 immunoreactivity in the SCWM (Figure 3 A and C). Intravenous MSC administration partially reduced IBA-1 immunoreactivity in the hippocampus, but the difference did not reach statistical significance (HI-SAL vs. HI-MSC; P=0.218) (Figure 3 B and D).

### MSC reduced white matter injury after global HI

The O4-positive preOLs observed in the SCWM were predominantly of immature phenotype (i.e. ring-shaped membrane staining, no processes) (Figure 4 A). The number of O4-positive cells was significantly (sham-SAL vs. HI-SAL; P<0.001) decreased after global HI in the SCWM (Figure 4 A and C), indicating HI-induced loss of preOLs in this region. Intravenous MSC administration significantly (HI-SAL vs. HI-MSC; P=0.002) increased the number of O4-positive preOLs with immature phenotype in the SCWM (Figure 4 A and C). The number of preOL was inversely related (*r* = -.83, P<0.001) to IBA-1 immunoreactivity in the SCWM (Figure S2 A).

Global HI significantly (sham-SAL vs. HI-SAL; P<0.001) reduced MBP immunoreactivity in the SCWM, indicating marked demyelination in this region (Figure 4 B and D). Accordingly, the number of MBP-positive mature oligodendrocytes clearly decreased following global HI (Figure 4 B). Intravenous MSC administration significantly increased (HI-SAL vs. HI-MSC; P=0.007) myelination (Figure 4 B and D). In line, loss of MBP-positive mature oligodendrocytes was prevented by MSC treatment (Figure 4 B). MBP immunoreactivity was inversely related (*r* = -.71, P=0.002) to IBA-1 immunoreactivity in the SCWM (Figure S2 B).

**Figure 5:**
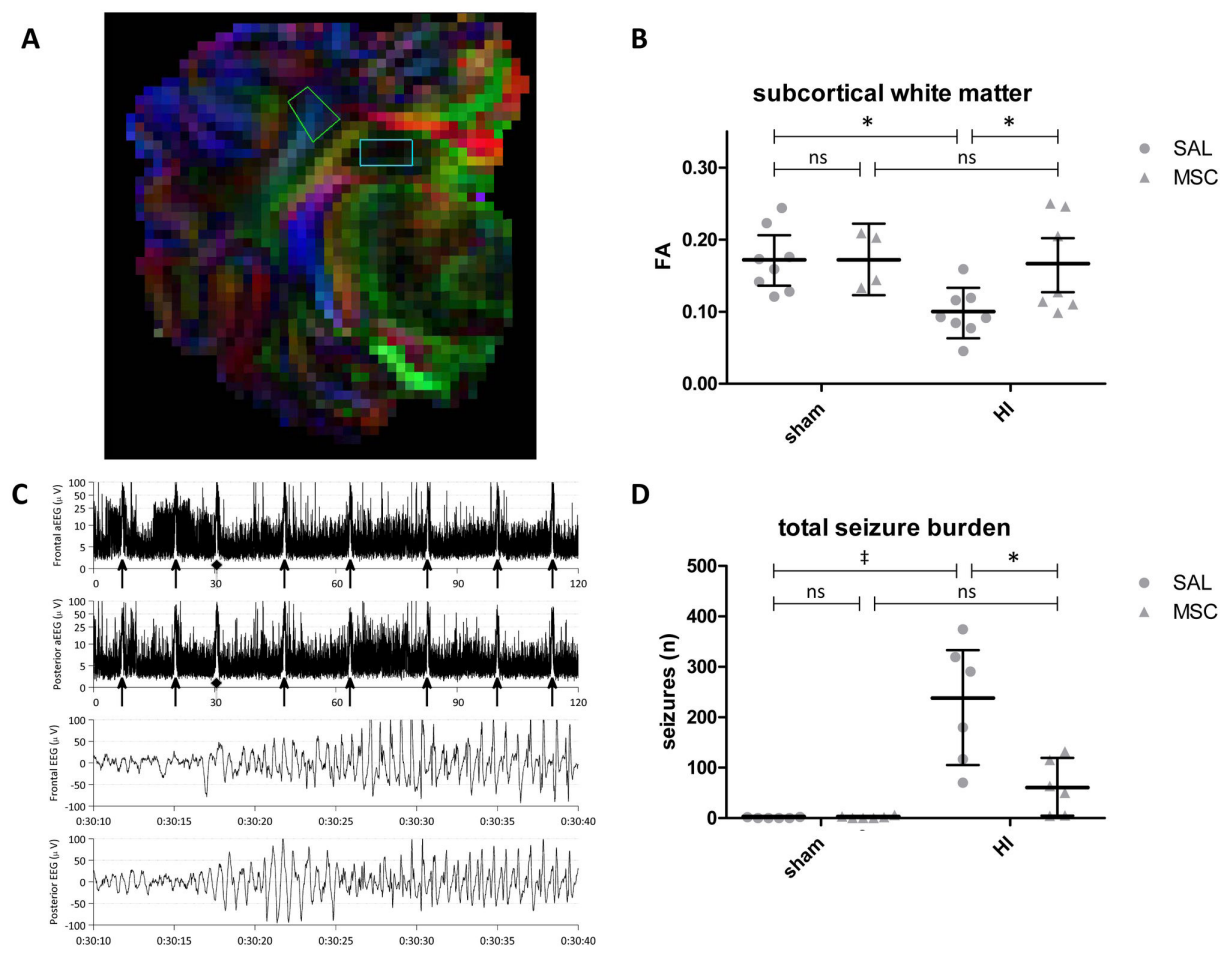
Intravenous MSC treatment prevented white matter injury and reduced electrographic seizure activity after global HI. (**A**) Regions of interest; SCWM and hippocampus in FA map. FA values in the hippocampus were not altered by global HI (data not shown). (**B**) Global HI caused a significant decrease in FA values in the SCWM, which was prevented by intravenous MSC administration; means ± 95% CI and levels of significance are depicted, which were calculated with ANOVA (sham-SAL n=8, sham-MSC n=4, HI-SAL n=8, HI-MSC n=8). Dots show each measurement (i.e. non-repeated measures) per animal. * *P*≤0.05, ‡ *P*≤0.01, # *P*≤0.001. FA = fractional anisotropy, SAL = saline, MSC = mesenchymal stem cells, HI = hypoxia-ischemia. (**C**) Representative image of an aEEG trace from a HI-SAL animal. The two upper traces represent aEEG traces of the C_3_-C_4_ and P_3_-P_4_ channel, respectively. The aEEG signal is time-compressed, showing a 120-min period. The amplitude is displayed semi-logarithmic, indicating a LMA of approximately 4-3 µV and UMA of approximately 10 µV. The arrows indicate seizure activity. The two lower traces depict the corresponding EEG signal (30 s) of one electrographic seizure activity indicated by the diamond. (**D**) Intravenous MSC administration significantly reduced electrographic seizure burden following global HI; medians ± IQR are depicted (sham-SAL n=6, sham-MSC n=6, HI-SAL n=6, HI-MSC n=6). * *P*≤0.05, ‡ *P*≤0.01, # *P*≤0.001. SAL = saline, MSC = mesenchymal stem cells, HI = hypoxia-ischemia.

FA values in the SCWM were significantly decreased (sham-SAL vs. HI-SAL; P=0.017) following global HI (Figure 5 A and B), indicating disturbed organization of white matter tracts. Intravenous MSC administration significantly increased (HI-SAL vs. HI-MSC; P=0.028) FA values in the SCWM following global HI (Figure 5 B), indicating that intravenous MSC prevented disturbances in white matter tracts organization. In line with clinical findings [33], DTI did not show significant changes of FA values after global HI in the hippocampus (data not shown).

### MSC reduced the electrographic seizure burden following global HI

Global HI significantly increased (sham-SAL vs. HI-SAL p=0.008) the electrographic seizure activity following UCO (Figure 5 C-D). In line with clinical [34] and experimental [35] data, the seizure activity peaked in the first 24 h after UCO, followed by a second smaller peak in the period between 24 and 48 h after UCO (data not shown). Intravenous MSC administration significantly reduced (HI-SAL vs. HI-MSC p=0.031) the number of electrographic seizures during the study period after global HI (Figure 5 D). Seizure activity was very rarely detected in sham animals treated with saline or MSC (Figure 5 D). Neuronal injury was further demonstrated by significant (sham-SAL vs. HI-SAL; P<0.001) brain atrophy following global HI (Table 1), which was diminished (HI-SAL vs. HI-MSC; P=0.058 and sham-MSC vs. HI-MSC; P=0.381) by intravenous MSC treatment (Table 1).

### MSC induced sustained T-cell tolerance

**Figure 6:**
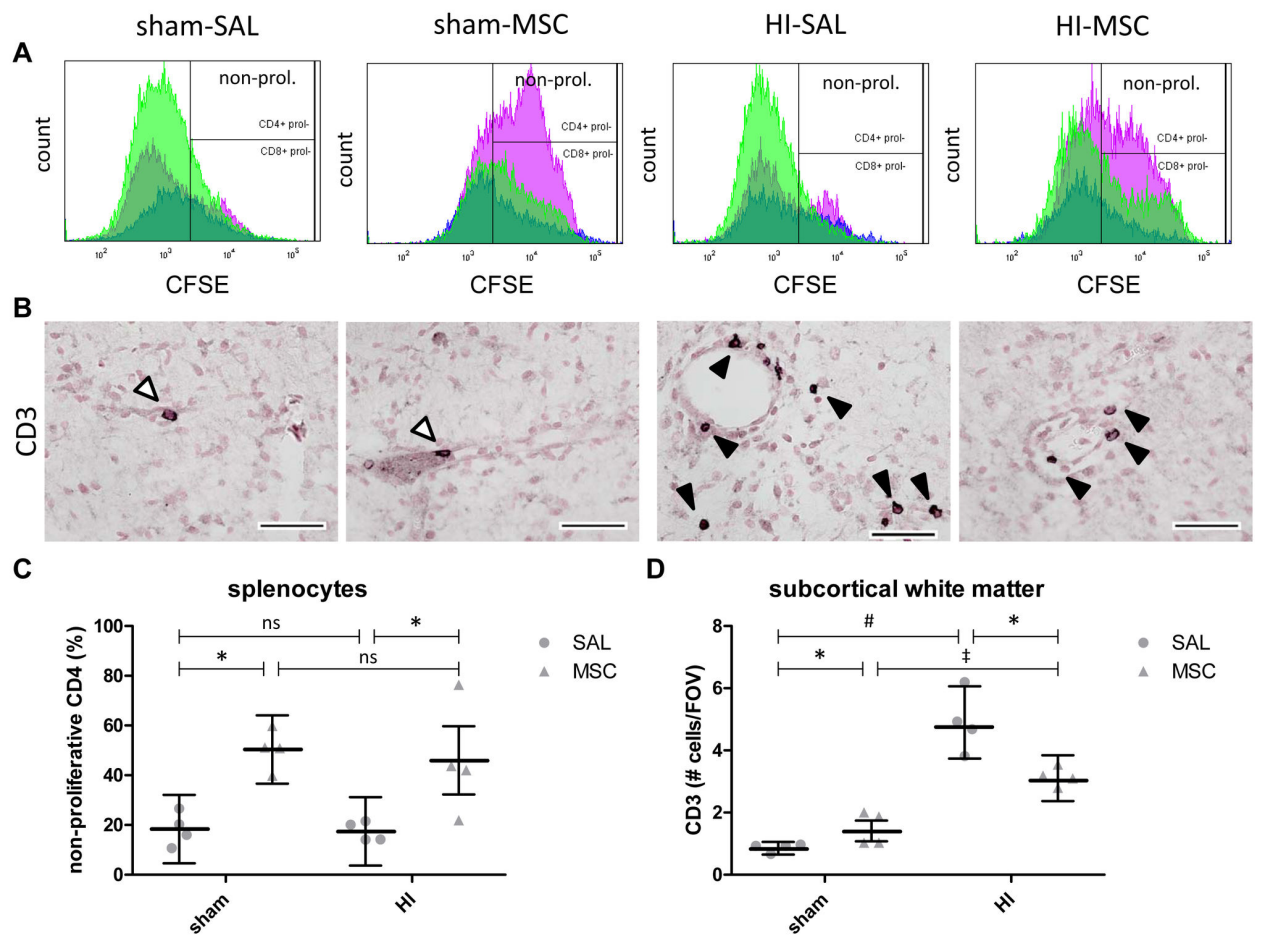
Intravenous MSC modulated the peripheral T-cell response after global HI. MSC persistently suppressed the proliferative capacity of CD4-positive splenocytes and reduced the number of invading T-cells into the preterm brain follow global HI and. (**A**) Representative flow cytometry histograms showing proliferation (increased CFSE dilution) of CD4-positive splenocytes (green) and CD8-positive splenocytes (blue) derived from the different experimental groups. Global HI did not alter the proliferative capacity of CD4-positive splenocytes (HI-SAL *versus* sham-SAL). MSC significantly suppressed the proliferation of CD4-positive splenocytes in sham operated animals (sham-MSC *versus* sham-SAL) and in animals exposed to global HI (HI-MSC *versus* HI-SAL). (**B**) Immunohistochemical CD3 (T-cell) staining in the subcortical white matter (SCWM) of all four experimental groups. In sham conditions, T-cells were primarily detected in the intravascular space (open arrows). Following global HI, a significant increase in the number of perivascular and interstitial (black arrows) CD3-positive cells was observed. T-cell invasion after global HI was significantly reduced by MSC. (**C**–**D**) Graphical presentation of (**C**) the percentage of non-proliferative CD4-positive splenocytes and (**D**) number of CD3-positive cells in SCWM; (**C**) means ± 95% CI and (**D**) geometric means ± 95% CI and levels of significance are depicted, which were calculated by ANOVA for CD4 proliferation (**C**) and the random intercept model with all repeated measures (i.e. brain sections) for CD3 counts (**D**). Dots show each measurement (i.e. non-repeated measures) per animal for CD4 proliferation (**C**) and the averaged results of the repeated measures (i.e. brain sections) per animal for CD3 counts (**D**). * *P*≤0.05, ‡ *P*≤0.01, # *P*≤0.001. SAL = saline, MSC = mesenchymal stem cells, HI = hypoxia-ischemia, FOV = field of view.

The percentage of non-proliferative (i.e. reduced CFSE dilution) CD4-positive splenocytes derived from HI-SAL animals was not significantly altered compared to those splenocytes from sham-SAL animals (Figure 6 A and C), indicating that global HI did not alter the proliferative capacity of splenocytes. Remarkably, the percentage of non-proliferative CD4-positive splenocytes derived from sham-MSC animals was significantly (P=0.012) increased compared to the sham-SAL group (Figure 6 A and C). In line, the percentage of non-proliferative CD4-positive splenocytes derived from HI-MSC animals was significantly (P=0.020) increased compared to the HI-SAL group (Figure 6 A and C), indicating that intravenously administered MSC persistently suppressed the *in vivo* responsiveness of CD4-positive helper T cells in the spleen. The proliferation capacity of CD8-positive splenocytes was not affected by global HI or MSC (data not shown). MSC-mediated suppression of helper T-cells *in vivo* was inversely related (Pearson *r* = -.60, P=0.035) to proliferation of microglia in the subcortical white matter (Figure S2 C).

### MSC reduced T-cell invasion after global HI

In sham conditions, T-cells were predominantly detected in the intravascular space of the preterm brain (Figure 6 B). Global HI significantly increased (sham-SAL vs. HI-SAL; P<0.001) the number T-cells in the SCWM (Figure 6 B and D). In the hypoxic-ischemic preterm brain, T-cells were primarily located in the perivascular and interstitial space, indicating HI-induced invasion of T-cells into the preterm brain (Figure 6 B). Remarkably, intravenous MSC administration significantly reduced (HI-SAL vs. HI-MSC; P=0.028) the number of T-cells in the SCWM following global HI (Figure 6 B and D), indicating that intravenous MSC prevented invasion of T-cells into the preterm brain.

### Detection of MSC in the preterm brain seven days after administration

**Figure 7:**
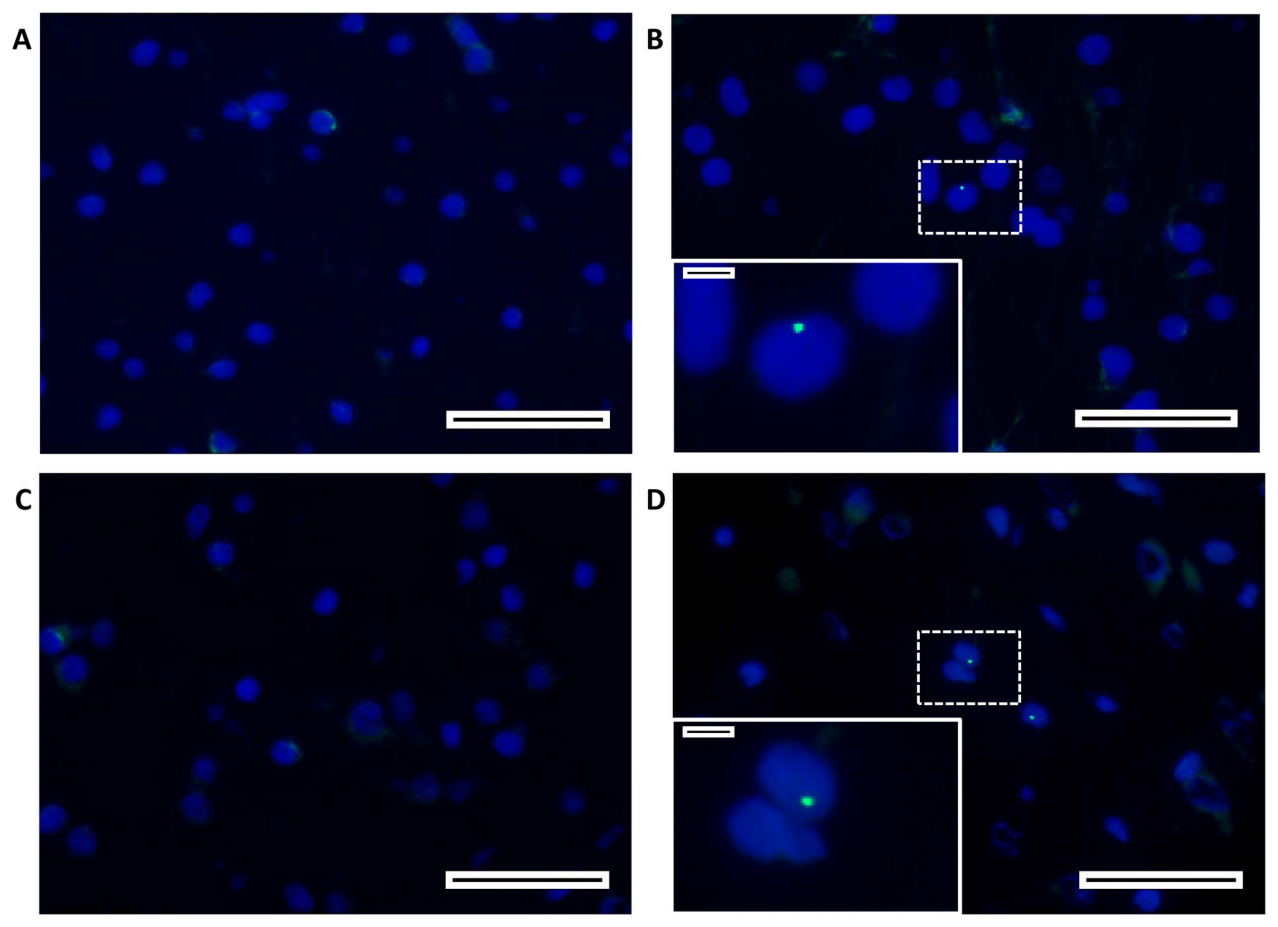
MSC were detected in the fetal ovine brain. A FISH probe specific for the human Y-chromosome detected the presence of systemically delivered MSC in the ovine brain. (**A**–**D**) Representative fluorescent images of the SCWM in the different experimental groups; (**A**) sham-SAL, (**B**) sham-MSC, (**C**) HI-SAL and (**D**) HI-MSC. (**A**–**B**) In saline treated animals the FISH probe did not react with any nucleus. (**C**–**D**) The FISH probe was detected in MSC treated animals indicating that MSC were present in the preterm brain 7 d after intravenous administration. Scale bars: (**A**–**D**) 50 µm, scale bars inserts: (**A**–**D**) 5 µm.

Within the subcortical white matter of MSC treated animals, we localized 20-40 cells per section that stained positive for the human specific Y-chromosome probe (Figure 7 C and D). MSC were detected in 4 of 5 studied animals that received MSC. The presence of MSC in the brain was confirmed by human-specific β2-microglobulin PCR (Figure S3). The amplified PCR products contained the correct human β2-microglobulin sequences as evaluated by sequencing. In addition, MSC were detected with PCR in SCWM, hippocampus, lung and spleen (data not shown).

## Discussion

Hypoxic-ischemic encephalopathy (HIE) in preterm infants is associated with cognitive and motor disorders, for which there is currently no therapeutic cure. The lack of feasible experimental models which are comparable to human neurodevelopment is one of the main hurdles that prevent the development of novel therapeutic strategies for hypoxic-ischemic injury in the preterm brain. We and others previously demonstrated that the fetal ovine model of global hypoxia-ischemia (HI) by umbilical cord occlusion (UCO) closely resembles the situation in human preterm infants in terms of neurodevelopment and white matter injury, which is the clinical hallmark of hypoxic-ischemic injury in the preterm brain [1,11,12]. In the current study, we use this translational animal model to provide evidence for the therapeutic potential of intravenous administration of MSC in the preterm brain after global HI.

We first assessed the effects on the cerebral anti-inflammatory effects of MSC by studying the proliferative response of microglia, which are typically activated early after global HI [11,36]. Our findings demonstrated that intravenous administration of MSC reduced the cerebral inflammatory response in the hypoxic-ischemic preterm brain after global HI. Secondly, we studied the protective effects of MSC on white matter injury. We showed that global HI induced a marked loss of preOLs, which was paralleled by hypomyelination of the preterm brain. In line with clinical evidence [37], FA values obtained with diffusion tensor imaging (DTI) were decreased following global HI indicating HI-induced breakdown of white matter organization. Intravenous administration of MSC prevented the loss of preOLs after global HI and reduced histological white matter injury, which was confirmed with DTI. The third step was to study neuronal injury on a functional level by assessing electrographic seizure burden following global HI in amplitude-integrated electroencephalogram (aEEG). Our findings showed that intravenous MSC treatment decreased the electrographic seizure activity following global HI. Reducing seizure activity is clinically highly relevant, since several studies have shown that seizures in neonatal HIE are associated with adverse neurodevelopmental outcome [38–40].

The preceding findings showed that intravenous MSC reduced the initial inflammatory response in the preterm brain mediated by microglia and provided protection against evolving white matter and neuronal injury after global HI. To further assess the mechanism underlying the therapeutic effects of MSCs, we studied modulation of the peripheral T-cell response and subsequent invasion of these cells into the ischemic preterm brain. We and others have previously shown that the initial cerebral inflammatory response after the hypoxic-ischemic insult is followed by a second peripheral inflammatory response characterized by mobilization and invasion of peripheral immune effector cells (i.e. neutrophils and T-cells) into the preterm brain [10,11,36,41]. Previous *in vitro* studies have demonstrated that MSC suppressed helper T-cell proliferation and induced an anti-inflammatory, more tolerant, phenotype in these immune effector cells [42–44]. These findings were confirmed and extended by our study showing that MSC induced persistent tolerance of T-cells *in vivo*. Furthermore, we showed that the degree of peripheral T-cell tolerance was inversely related to cerebral inflammation. Moreover we found that MSC reduced T-cell invasion following global HI. Based on these findings, we hypothesize that sequestered MSC modulated peripheral helper T-cells to become unresponsive, making them less susceptible for activation and mobilization in response to brain-derived damage signals following global HI. This suppression of peripheral immune cells may decrease invasion of immune effector cells into the brain observed after global HI, thereby reducing a second inflammatory hit to the ischemic preterm brain [11].

The newly identified modulation of T-cell tolerance *in vivo* and reduction of T-cell invasion after intravenous MSC administration adds to the understanding of the anti-inflammatory effects of MSC in the context of inflammation-driven organ injury. Besides the anti-inflammatory effects of MSC in our model, we speculate that the observed protective effects of MSC on white matter injury are based on additional mechanisms. First, the MSC-mediated reduction of cerebral inflammation potentially reduced injury to vulnerable preOLs in the preterm brain. This concept is supported by studies showing microglia-mediated injury to preOLs [45] and our findings that preOL density and myelination in the subcortical white matter were inversely related to proliferation of microglia. A second effect might be attributed to MSC-mediated regeneration of lost preOLs and diminished arrested oligodendrocyte maturation. The latter concept is supported by *in vitro* data showing that MSC stimulate neural progenitor cells to differentiate towards the oligodendrocyte lineage [46,47] and *in vivo* data demonstrating remyelination after intracerebral delivery of MSC in a rat model of neonatal hypoxic-ischemic brain injury [48].

From the MSC that were administered intravenously, only a few cells (<0.01%) were located within the preterm ovine brain at seven days after global HI. PCR and sequencing confirmed the presence of MSC in the brain and also confirmed that MSC were present in lung and spleen seven days after intravenous administration. In concordance with our findings, a study by Lee et al. showed that MSC were neuroprotective in a rat model of neonatal hypoxic-ischemic brain injury, while less than 0.01% of intravenously administered MSC were recovered in brain, lung and spleen tissue within 96 h after infusion [49]. Remarkably, also intracerebral injected MSC were shown to be neuroprotective, although they disappeared rapidly after injection [50]. These findings suggest that the neuroprotective effects of MSC last longer than their presence implying that MSC-mediated modulation of inflammation and repair of injury is initiated early after administration.

Several other studies using rodent models of neonatal hypoxic-ischemic brain injury have previously shown neuroprotective effects of MSC after intracerebral injection [18,20,48], intranasal [51] and intravenous administration [17,21]. These rodent models however have several limitations that make them less suitable for clinical translation [23,24,52]. The strength of the instrumented preterm sheep model is that it closely resembles human preterm neurodevelopment and second, that the global HI insult induced by umbilical cord occlusion accurately mimics the common clinical etiology of HIE in preterm infants [24,52].

We showed that xenotransplanted human MSC effectively modulated immune responses and stimulated neuronal repair in preterm sheep, without any signs of immunological rejection. These findings are in line with early work by Zanjani et al. showing that human xenotransplanted HSC were well tolerated by the permissive environment of preimmune fetal sheep [53] and even after establishment of the fetal immune system [32]. The latter findings indicated that MSC have unique immunological characteristics that allow them to persist in a xenogeneic environment [32].

The observed neuroprotective effects in our model were seen following severe hypoxic-ischemic brain injury suggesting that the MSC-mediated treatment effects will be enhanced following milder episodes of ischemia in the preterm brain. Although the dosage of MSC administered in our model was consistent with completed [21,54] and ongoing (ClinicalTrials.gov: NCT01501773, NCT01297413, NCT01678534) clinical trials of focal cerebral ischemia (i.e. stroke), dose escalation studies need to be conducted to determine the optimal dosing strategy in hypoxic-ischemic preterm brain injury. In addition, the magnitude of the treatment effect may depend on the timing of intervention; we infused MSC 1 h after the HI insult particularly aimed at intervening early with the detrimental inflammatory response following global HI. However, it is conceivable that repetitive dosing may especially enhance the regenerative effects of MSC. Furthermore, neuroprotective effects of MSC observed here may have been underestimated after a reperfusion period of one week. It is plausible that additional MSC-mediated repair of white matter and neuronal networks takes place beyond the period of one week after global HI. Whether the observed structural changes and reduction in electrographic seizures are associated with long term improvement of cognitive and motoric function needs to be addressed in future (clinical) studies [13].

In conclusion, we report for the first time that intravenously administered mesenchymal stem cells (MSC) were neuroprotective in a translational ovine model of preterm brain injury after global HI. We demonstrated that MSC protected the preterm brain against white matter injury and established functional improvement. We provide evidence that the neuroprotective effects of MSC may be mediated by modulation of the peripheral T-cell response after global HI. Further experimental research is needed to elucidate the anti-inflammatory and regenerative mechanisms of action of MSC and to evaluate the long-term therapeutic effect of MSC on motoric and cognitive function after global HI. Future clinical trials should focus on the optimization of timing and dosing of MSC therapy in preterm infants with hypoxic-ischemic brain injury.

## Supporting Information

Figure S1 **Gating strategy proliferation assay.**
(**A**–**C**) Dot plots illustrating gating strategy in the flow cytometry analysis of the proliferation assay. FSC = forward scatter, SSC = sideward scatter, 7-AAD = 7-Aminoactinomycin D (viability stain).
(TIF)

Figure S2 **Correlation plots.**
(**A**) Density of immature preOLs was inversely related (Pearson *r* = -.83, P<0.001) to IBA-1 immunoreactivity in the SCWM. (**B**) MBP immunoreactivity was inversely related (Pearson *r* = -.71, P=0.002) to IBA-1 immunoreactivity in the SCWM. (**C**) The number of non-proliferating CD4-positive T cells harvested from the spleen was inversely related (Pearson *r* = -.60, P=0.035) to IBA-1 immunoreactivity in the subcortical white matter.
(TIF)

Figure S3 **Detection of human specific β-2-microglobulin DNA sequences in preterm sheep brain.**
Genomic DNA was extracted from subcortical white matter of a MSC treated animal (MSC) and a saline treated animal (SAL) and analyzed by nested PCR for the presence of human β-2-microglobulin (B2M) DNA sequences. The presence of amplifiable DNA was evaluated by PCR for β-actin (actin). - = water control, + = positive control; genomic DNA extracted from one million human MSC.
(TIF)
