# Supplementary figures and images for: Mesenchymal Stem Cells Induce T-Cell Tolerance and Protect the Preterm Brain after Global Hypoxia-Ischemia
Source: PLoS One. 2013 Aug 26;8(8):e73031. doi: 10.1371/journal.pone.0073031 (PMC3753351; doi:10.1371/journal.pone.0073031)

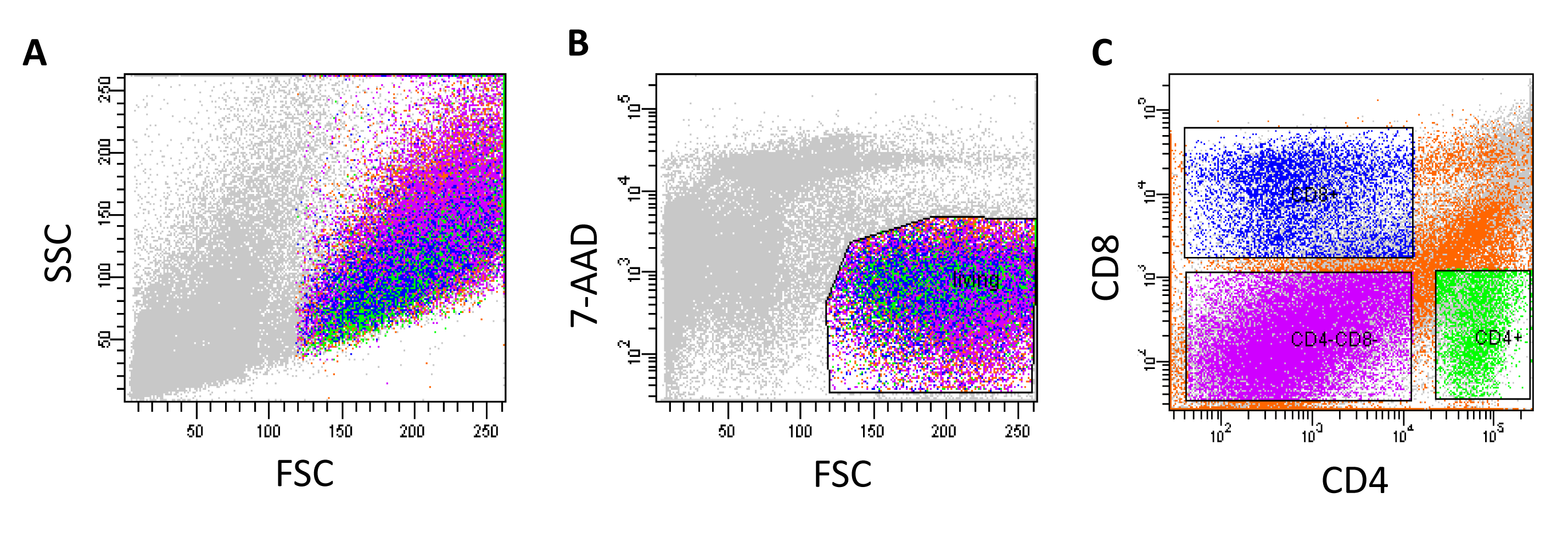

Supplement: Figure S1 — Gating strategy proliferation assay. (A–C) Dot plots illustrating gating strategy in the flow cytometry analysis of the proliferation assay. FSC = forward scatter, SSC = sideward scatter, 7-AAD = 7-Aminoactinomycin D (viability stain). (TIF) [file pone.0073031.s001.tif]

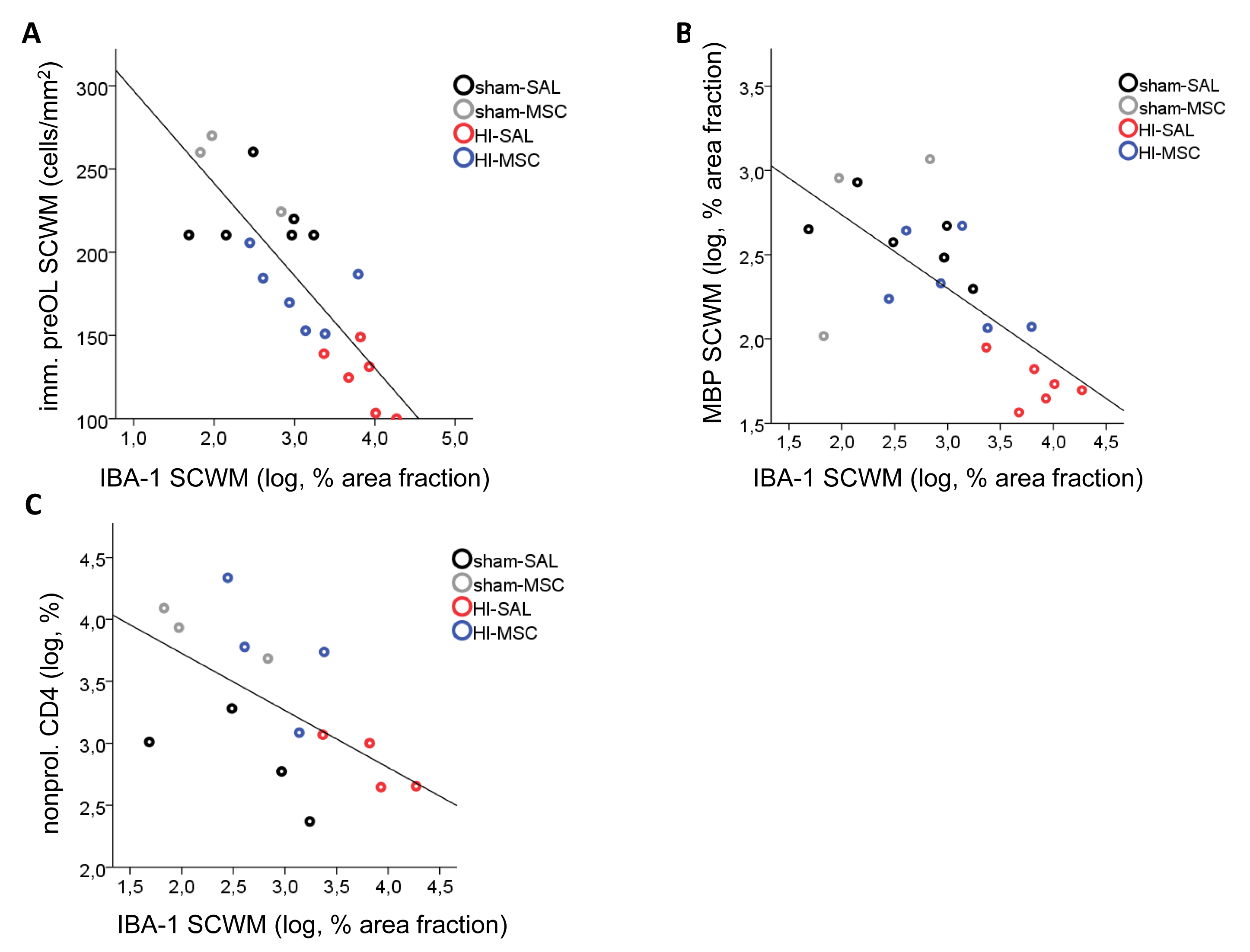

Supplement: Figure S2 — Correlation plots. (A) Density of immature preOLs was inversely related (Pearson r = -.83, P<0.001) to IBA-1 immunoreactivity in the SCWM. (B) MBP immunoreactivity was inversely related (Pearson r = -.71, P=0.002) to IBA-1 immunoreactivity in the SCWM. (C) The number of non-proliferating CD4-positive T cells harvested from the spleen was inversely related (Pearson r = -.60, P=0.035) to IBA-1 immunoreactivity in the subcortical white matter. (TIF) [file pone.0073031.s002.tif]

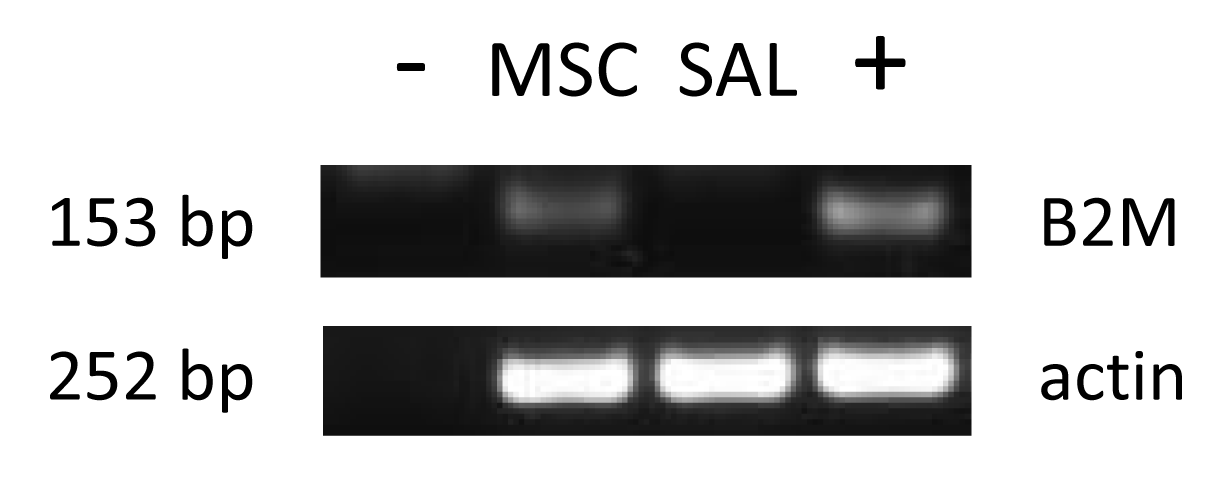

Supplement: Figure S3 — Detection of human specific β-2-microglobulin DNA sequences in preterm sheep brain. Genomic DNA was extracted from subcortical white matter of a MSC treated animal (MSC) and a saline treated animal (SAL) and analyzed by nested PCR for the presence of human β-2-microglobulin (B2M) DNA sequences. The presence of amplifiable DNA was evaluated by PCR for β-actin (actin). - = water control, + = positive control; genomic DNA extracted from one million human MSC. (TIF) [file pone.0073031.s003.tif]
